# Systemic Ammonia Toxicity: An Underestimated Driver of Cerebral Energy Crisis in Hepatic Encephalopathy

**DOI:** 10.3390/ijms27156739

**Published:** 2026-07-28

**Authors:** Lyudmila Tikhonova, Eugene Maevsky, Carmina Montoliu, Elena Kosenko

**Affiliations:** 1 Institute of Theoretical and Experimental Biophysics of Russian Academy of Sciences, 142290 Pushchino, Russia; ljudasik09@rambler.ru (L.T.); eim11@mail.ru (E.M.); 2 INCLIVA Biomedical Research Institute, 46010 Valencia, Spain; 3 Department of Pathology, Faculty of Medicine, University of Valencia, 46010 Valencia, Spain

**Keywords:** hepatic encephalopathy, ammonia, liver, erythrocytes, brain, interorgan metabolic communication

## Abstract

Hepatic encephalopathy (HE) is a complex of pathological processes in the brain caused by liver failure or portosystemic shunting. Even though ammonia (In this review, the term “ammonia” refers to total ammonia (ammonia gas and ammonium ion)) is widely recognized as the primary neurotoxin responsible for triggering the cerebral energy crisis and subsequent neurological manifestations of HE, its broader systemic effects are often overlooked. Meanwhile, the brain, which features the highest level of oxidative metabolism and extremely low energy reserves requires a constant supply of highly oxygenated and glucose-rich blood. Therefore, ammonia-induced disruptions in interorgan metabolic communication, leading to a restriction of vital energy substrates reaching the brain, are highly likely involved in this pathology. Currently, ammonia-related impairment of the metabolic relationship between the brain and extracerebral tissues is underestimated. This review summarizes generally accepted concepts and focuses on recent advances detailing how ammonia pathologically disrupts the highly integrated metabolic pathways in the liver and erythrocytes, thereby impairing the delivery of vital energy substrates to the brain. Additionally, the role of glutamate NMDA receptors in these metabolic disorders is discussed. The gathered information provides a deeper understanding of the indirect mechanisms by which ammonia compromises brain energy homeostasis, thereby ultimately leading to encephalopathy. Simultaneous measurement of plasma and erythrocyte ammonia is required to avoid measurement artifacts.

## 1. Introduction

Hepatic encephalopathy (HE) is a complex of pathological processes in the central nervous system leading to progressive neurological and mental illness in patients with severe liver disease or portosystemic shunt [1]. Due to the presence of multiple causes of brain pathology, the etiology of HE is not completely understood. According to numerous evidences, the mechanism of the brain injury is multifactorial and ammonia is a key postulated neurotoxin triggering a cascade of pathological reactions responsible for toxic-metabolic brain damage and clinical manifestations of HE [2]. However, it is well established that both forms of ammonia presented in the blood (NH_3_/NH_4_^+^) readily cross the membranes of both neuronal and non-neuronal cells [3]. Consequently, when blood ammonia levels are abnormally elevated (hyperammonemia, HA), this toxin causes systemic toxicity leading to multiple organ failure, rather than solely direct brain damage [4,5]. Furthermore, this systemic disruption impairs interorgan metabolic communication, restricting the production and delivery of vital energy substrates to the brain [6]. This limitation is particularly critical because the brain, unlike other organs, exhibits an exceptionally high rate of oxidative metabolism to support its myriad of functions and, having extremely low energy reserves, strictly requires a constant supply of highly oxygenated and glucose-rich blood [7,8].

In the case of an inadequate supply of blood glucose to the brain, the only biochemical pathways that maintain the normal endogenous glucose levels in the cerebral tissues are glycogenolysis and gluconeogenesis (GNG). At the same time, glycogen stores are limited and the rate of de novo glucose synthesis in the brain is extremely low [9]. When the brain’s demand for energy substrates is not met by endogenous resources, normoglycemia is achieved through the release of glucose from the liver as a result of glycogenolysis and the GNG pathway, which produces glucose “de novo” using non-carbohydrate substrates.

Like glucose, ketone bodies produced by the liver are also released into the bloodstream, and serve as additional energy sources for the brain [10]. Thus, the obligatory dependence of the vital activity of the brain on the metabolic function of the liver is axiomatic. Unfortunately, despite extensive research into the mechanisms of HE, the literature on ammonia-induced disturbances of liver metabolic pathways (except for the urea cycle) remains fragmented. This scarcity of data persists because it is generally assumed that the liver, being evolutionarily adapted for ammonia detoxification, cannot be damaged by the toxin itself [11,12]. However, this traditional view fails to account for the systemic nature of HE, where ammonia acts as a multiorgan toxin that directly impairs the hepatic metabolic capacity itself [3,6]. Therefore, to identify the true mechanisms of this pathology, a comprehensive analysis of recent advances is crucial. Understanding how ammonia derails highly integrated metabolic pathways in the liver, thereby disrupting blood glucose homeostasis, will help clarify how it leads to an energy crisis in the brain and, ultimately, to encephalopathy.

Another missing link in understanding the causes of hepatic encephalopathy is the underestimation of the role of erythrocytes, key components of the global transport system [13], which directly deliver oxygen to tissues and maintain energy homeostasis [14,15], necessary for cellular vital activity. As a rule, the main information about the disruption of oxygen delivery to the brain is obtained mostly from the results of measuring cerebral blood flow (CBF) and the cerebral metabolic rate of oxygen (CMRO_2_) [16,17]. However, due to the activation of various endogenous autoregulatory factors in each specific case [18,19], the final results of the measurement of these indicators can be ambiguous [20]. In addition, to assess the efficiency of oxygen transfer from the lungs to the tissues, the amount of oxygen in the whole blood is usually measured as arterial blood oxygen saturation (SaO_2_) and partial pressure of oxygen (PaO_2_) [21]. Surely, the presence of oxygen in the blood is an essential factor but it is not sufficient. In this context, it is necessary to elucidate whether the hemoglobin (Hb) of erythrocytes, having bound even the maximum possible amount of oxygen in the lungs, is capable of unhindered release of the required amount of oxygen to the tissues during gas exchange, which is determined by the Hb-oxygen affinity (Hb-O_2_ affinity) [22]. The diversity of Hb-O_2_ affinity (high in the lungs and low at the site of gas exchange), in turn, depends on the metabolic pathways of the erythrocytes controlled by biochemical processes that occur in the membrane structure and inside the erythrocytes [23]. To date, in clinical settings, Hb-O_2_ affinity is still, however, estimated only by the oxygen dissociation curve (ODC) plotted under artificial conditions using standard indices (pH: 7.4, PCO_2_: 40 mmHg, temperature: 37 °C) [24,25]. Undoubtedly, this artificial normalization fails to account for the abnormal cellular environment and potential pathological shift in seriously ill patients, thereby misrepresenting their actual in vivo Hb-O_2_ affinity. In addition, many factors that can unpredictably alter the ODC are often overlooked. In particular, they are erythrocyte endogenous allosteric modifiers of Hb-O_2_ affinity [26] whose levels vary across different diseases [27] and depend on age, gender [28], genetics [29], ethnicity [30], and other biological characteristics [31]. Consequently, it leads to conflicting results and erroneous conclusions [32].

Furthermore, the above traditional methods often neglect key erythrocyte-derived metabolic byproducts that regulate not only Hb-O_2_ affinity, but also NO-dependent vasodilation [33], capillary blood flow [14], endothelial function [33], blood rheology, and systemic metabolic homeostasis [34].

Similarly, while increased red blood cell distribution width (RDW) reliably reflects the impaired biochemical and morphological status of erythrocytes, its broader diagnostic significance remains underappreciated. RDW is still an extremely important indicator in the diagnosis of hemolytic anemia [35,36] and an independent marker of disease progression and mortality [37]. Since increased RDW is a consequence of metabolic/energetic defects of erythrocytes, it becomes obvious that a loss of rheological properties of these cells, which leads to a decrease in oxygen supply to tissues [23], can occur long before the onset of hemolysis [38,39].

And finally, in spite of the fact that the toxic effect of ammonia on erythrocytes, accompanied by their swelling and lysis, was discovered more than a century ago [40] and confirmed by a number of studies [41,42,43,44], these fundamental observations have not yet received the attention they deserve. As of today, the erythrotoxic potency of ammonia, the concentration of which in erythrocytes of patients with liver disease is always several times higher than in plasma [45,46], is still not considered, even while revealing the mechanisms of hemolytic anemia associated with liver cirrhosis.

Overstimulation of NMDA receptors (NMDAR), recently discovered in erythrocytes, can be another highly detrimental factor. NMDAR hyperactivation has been repeatedly confirmed to play a key role in the ammonia-induced cascade affecting multiple oxygen-dependent metabolic pathways [47,48,49] in the brain [50,51], leading to an energy crisis and neurological manifestations. In particular, it has been shown that ammonia accumulation in the brain slows down the flow of electrons in the mitochondrial respiratory chain. This, in turn, leads to impaired oxidative phosphorylation and ATP production [47], disruption of Ca^2+^ homeostasis [48] and a significant increase in the level of reactive oxygen and nitrogen species [52], reduces the activity of antioxidant enzymes and glutamine synthetase, which leads to oxidative stress [53], and also slows down ammonia detoxification in the brain and is lethal to animals [54]. However, the involvement of these receptors in erythrocyte damage under conditions of ammonia intoxication remains poorly understood. Data on the ammonia levels that can be achieved in erythrocytes in HA (except for a few aforementioned early reports) is also currently scarce.

And consequently, the role of aberrant metabolic-energy processes in erythrocytes of disrupting their oxygen transport function has not received enough attention at present, and is not a diagnostic tool for the “disease” of the erythrocytes themselves. Hence, the power of basic science aimed at identifying early markers that may precede the development of multiorgan hypoxia observed in many diseases accompanied by encephalopathy is also undervalued [55,56,57,58,59,60,61].

To bridge the gap in the understanding of the indirect mechanisms by which ammonia compromises brain energy homeostasis, this review comprehensively evaluates how effects of ammonia on both metabolic pathways in the liver and the oxygen-carrying function of erythrocytes contribute to the disruption of interorgan metabolic crosstalk, leading to HE.

## 2. Ammonia: Neuro- or Hepatotoxin

Despite the constant formation of ammonia in the body, its concentration in blood plasma under physiological conditions is maintained at a very low level and does not exceed 50 μmol/L [12]. This is achieved exclusively through its detoxification in the liver. Initially, ammonia and glutamine (the transport form of ammonia), formed in tissues and the digestive tract (detailed information is provided in [62,63,64]) accumulate in the hepatic portal vein and then enter the periportal hepatocytes, which contain a complete set of enzymes necessary for ammonia detoxification in the urea cycle.

Therefore, it is generally accepted that elevated blood ammonia (hyperammonemia, HA) occurs whenever hepatocytes lose the ability to synthesize urea in case of cirrhosis or when blood is shunted past the liver [1,65]. In addition, it has been postulated that it is systemic ammonia that is the main neurotoxin causing functional disorders exclusively of the nervous system and clinical manifestations of HE [2]. Herewith, it is also believed that the liver function, which is evolutionarily related to complete neutralization of ammonia, cannot itself be disturbed by ammonia [11,12]. It has been found, however, that this “neurotoxin” also causes multiorgan damage, including to the liver [3,66]. Although compelling evidence to support these observations is still relatively limited, the available data suggest that the generally accepted role of systemic ammonia solely as a neurotoxin in the pathogenesis of HE cannot be viewed as an axiom. To understand the role of ammonia towards damage of the liver, it is necessary to analyze the possible causes of ammonia accumulation in the liver and its effect on hepatocytes.

Some biochemical processes are considered below, where disruption might allow ammonia to “pass through” the urea cycle and, consequently, accumulate in the liver cells and then not only be released into the systemic circulation but also elicit toxic effects on hepatocytes.

### Possible Causes for the Hepatotoxic Effects of Ammonia

Carbamoyl phosphate synthetase (CPS I), the first enzyme that initiates the urea cycle, is localized in the mitochondria of periportal hepatocytes and is the rate-limiting enzyme and has a relatively high Km (2–3.5 mM) for ammonia [67]. This means that activation of CPS I requires a significant amount of ammonia, which is available by the presence of ammonia that fluxes via the portal vein to the liver and the one additionally formed from glutamine under the action of hepatic phosphate-dependent glutaminase [65]. At the same time, although local ammonia concentration in periportal hepatocytes significantly exceeds that in the systemic circulation, and some part of the ammonia usually passes by the urea cycle “unnoticed” and may diffuse away [67], under physiological conditions ammonia neither has a toxic effect on the liver nor releases in large quantities into the general circulation. This occurs due to additional neutralization of ammonia through conversion to glutamine in a reaction catalyzed by glutamine synthetase (GS), localized in the perivenous zone of the liver [67]. However, these physiological processes fail when ammonia entering the liver in excessive quantities disrupts the regulatory process that ensures the interaction of the urea cycle and GS [68,69]. As a result, ammonia detoxification by GS slows down thereby promoting additional accumulation of ammonia in liver cells [68,70]. This, in turn, leads to activation of hepatic ammonia-metabolizing reactions, resulting in the formation of negative effectors that further inhibit GS [71,72] and/or enhance glutaminase activity [73]. Thus, in the liver, ammonia blocks its own conversion to glutamine thereby creating background for a vicious circle that aggravates ammonia-associated hepatic injury and inevitably worsens HA.

Since HA may arise from a direct toxic effect of ammonia on the liver unrelated to liver disease or portosystemic shunting [12,74,75], this suggests that the role of ammonia postulated only as a neurotoxin is incomplete and besides the impact on the brain, ammonia deteriorate status of other organs, including the liver itself.

This suggestion is further supported by findings showing that ammonia accelerates the formation of fibrous tissue [76,77]. This, in turn, contributes to more profound disturbances of metabolic pathways in hepatocytes, particularly of those affecting systemic glucose homeostasis [78], the disruption of which indicates the liver’s inability to produce vital metabolites that ensure normal brain function [79,80].

However, the role of ammonia-induced disturbances of hepatic metabolic pathways in the development of fatal cerebral failure in cases associated (or not associated) with primary hepatocellular dysfunction remains poorly understood and is rarely considered in combination with known causes of HE. Therefore, the following section summarizes the available literature data explaining how ammonia-related perturbations in the metabolic capacity of liver cells may be associated with brain pathology.

## 3. Dysregulation of Glucose Homeostasis as a Key Factor of Ammonia-Related Changes in Liver Metabolic Function: Controversies and Limitations

Glucose is the major fuel for the brain and a sharply reduced level of glucose in the blood (hypoglycemia) is thus one of the most important risk factors for the development of brain disorder [81]. Although hypoglycemia with fatal consequences, observed in the past in a relatively small group of patients with or without liver disease [82], has now been confirmed by numerous studies [83,84,85], the specific mechanisms responsible for this pathology have not been definitively established.

As noted, the liver plays a unique and irreplaceable role in maintaining blood glucose homeostasis through various carbohydrate metabolic pathways. On the whole, it is assumed that disturbances in the glucose-producing pathways can occur in liver diseases only at the final, fibrous stage when a normal structure of the most liver cells is disrupted, making them unable to detoxify ammonia and contributing to a sharp accumulation of this toxin in the liver [86].

However, it is difficult to gather direct evidence from the limited clinical studies in this area as to whether ammonia is involved in the disruption of hepatic glucose production pathways. Rather contradictory in vitro findings obtained using perfused animal liver or hepatocytes do not shed light on the influence of ammonia on this process either [87,88,89]. A discussion of the available data concerning this problem follows.

### 3.1. Possible Role of Ammonia in the Impairment of Hepatic Glycogenolysis: Remaining Questions

Compensatory formation of glucose in the liver occurs through two different pathways: glycogenolysis (glycogen breakdown) and GNG, the de novo synthesis of glucose from non-carbohydrate substrates. Whenever glucose content in the blood is greatly reduced, glycogen in the liver is quickly broken down into free glucose that is subsequently released into the blood, thereby maintaining the glucose level in the general circulation close to the physiological norm. A more comprehensive description of these physiological processes can be found in [90,91,92,93].

Alterations in glycogen metabolism in the liver associated with the impairment of its postprandial synthesis, leading to restriction of glycogen stores and/or its inability to break down to form free glucose, have been described both in animal models [94], and humans with liver cirrhosis [95,96,97]. However, the mechanisms responsible for reducing glycogenic flux in this pathology have not been fully elucidated, and only a few factors have been suggested to explain this anomaly. Among them are diminished glucokinase activity [95] due to limited ATP availability [98], hormonal imbalances associated with a chronically decreased insulin/glucagon ratio [99], as well as a decrease in total and free insulin-like growth factors. Systemic ammonia that accelerates the formation of fibrosis contributing to the destruction of structural organization [77] of the liver cells can presumably be a factor in reducing the production of glucose in the liver during glycogenolysis [100]. Unfortunately, the identified mechanisms of the disruption of hepatic glycogen metabolism do not answer the question of whether ammonium has a direct effect on this pathway. Currently, only in vitro data show that human and animal hepatocytes are highly sensitive to ammonia toxicity. In particular, it has been shown that in primary human hepatocytes [101] and liver stellate cells, ammonia disturbs mitochondrial energy metabolism and promotes oxidative stress [86], which is responsible not only for the metabolic dysfunction of these cells but also for the inability of damaged cells to regenerate [102].

Furthermore, ammonia inhibited glycogen synthesis from glucose in hepatocytes from fasted rats [103] and isolated perfused rat liver [98]. Taken together, this suggests that human and animal hepatocytes in vitro are supposed to be the primary target of ammonia’s toxic effects. This may make liver cells unable to be engaged in glucose’s uptake and release into the bloodstream. However, extrapolation of the results obtained from the experiments performed in vitro to in vivo situations may be difficult for obvious reasons [104]. And hence, the question as to what the role of ammonia is in the impairment of glycogen metabolism in the liver, which may be an early and critical step in the development of ammonia-induced hepatotoxicity preceding hypoglycemia in patients without (or with) hepatocellular disease, remains open. The answer to this question requires future research.

### 3.2. Gluconeogenesis

Since impaired hepatic glycogen metabolism has been documented in most patients with liver cirrhosis, it has been suggested that to maintain normal hepatic glucose production and normoglycemia in these conditions, the rate of GNG needs to be increased [105]. However, early and current research results and conclusions regarding the greater contribution of GNG to maintenance of normoglycemia are highly controversial. Indeed, some research using different technical approaches [106] have shown that the rate of GNG may be normal in cirrhosis [107], while other researchers have suggested that after overnight fasting, the GNG rate increases [96] not only in patients with early stages of well-compensated cirrhosis [105] but also in cases of extensive fibrosis [100]. Apparently, the increase in GNG capacity in the initial stage of the disease can be considered as an adaptive process that helps to compensate for the depletion of glycogen stores and maintain normal glucose production by the liver, thereby preventing hypoglycemia. But the underlying reasons for the increased GNG in the development of fibrosis, accompanied by destruction of structural organization of the liver, chronic inflammation, breach of energy metabolism, oxidative stress, necrosis, cirrhosis and progressive organ failure [108] are still obscure.

**Figure 1:**
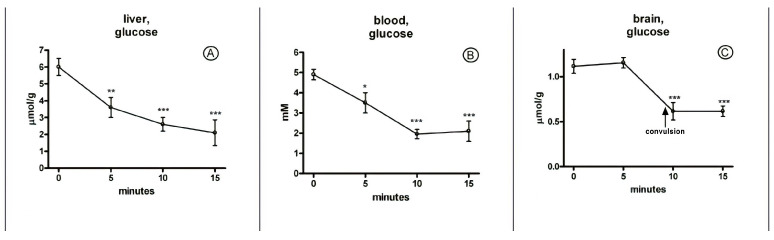
Time course of changes in glucose in the liver (**A**), blood (**B**), brain (**C**) in acute ammonia intoxication. Values are the means ± SEM (*n* = 8). Significant differences between the values with respect to the time point of 0 min were estimated by ANOVA analysis and corrected with Bonferroni: * *p* < 0.05, ** *p* < 0.01, *** *p* < 0.001. This figure is taken from an original article “A Look into Liver Mitochondrial Dysfunction as a Hallmark in Progression of Brain Energy Crisis and Development of Neurologic Symptoms in Hepatic Encephalopathy” by Kosenko E. et al., published in J. Clin. Med. 2020, 9, 2259; doi:10.3390/jcm9072259 [6].

Moreover, the main enhancers of GNG that maintain fasting normoglycemia in patients with advanced fibrosis are considered to be chronically elevated plasma glucagon concentrations [109] and hepatic insulin resistance [110], which through different mechanisms enhance the activity of regulatory enzymes and availability of gluconeogenic substrates [111]. However, this assumption cannot answer the question as to why in most patients at an advanced stage of fibrosis, during which the liver ability to synthesize glucose de novo may be compromised [112], neither normoglycemia nor hyperglycemia but severe hypoglycemia is observed [83]. Surely, causes for the development of hypoglycemia are multi-factorial and interrelated [85]. Nevertheless, it has long been convincingly proven that inhibition of GNG may play an increasingly more important role in the development of hypoglycemia [113]. It is therefore plausible that it is just this pathology that is responsible for symptoms of central nervous system dysfunction [114] and adverse clinical outcomes observed in patients with decompensated liver cirrhosis [84]. However, significant variations in reported data and contradictory conclusions regarding the rate of GNG complicate the assessment of its role in blood glucose homeostasis in patients with liver failure and encephalopathy. Furthermore, previous studies have largely overlooked the role of ammonia as the primary neurotoxin implicated in HE in both liver disease [2,11] and non-liver-related failure [75,115]. Consequently, the precise mechanisms by which HA induces encephalopathy need to be fully clarified. Data from animal models of hyperammonemia may elucidate this phenomenon. Although research remains sparse, in vivo studies demonstrate that acute ammonia intoxication inhibits hepatic GNG and disrupts blood glucose homeostasis, thereby compromising normal brain function. Thus, our previous and present findings demonstrate that in rats with acute HA, ammonia rapidly accumulates in liver mitochondria leading to cytochrome oxidase inhibition, oxidative phosphorylation disruption, and a sharp decrease in ATP concentration. In addition, it was found that ammonia sharply stimulates NMDAR-dependent formation of NO^•^ and superoxide radicals and a decrease in the activity of antioxidant enzymes leading to oxidative stress [5,41,116]. These changes, together with the identified negative energy balance and impaired redox potential, implied inhibition of hepatic GNG. Indeed, despite the presence of hyperglucagonemia in the fasted rats and elevated levels of intramitochondrial acetyl-CoA [117], the major intracellular activator of GNG, ammonia loading to the animals quickly and dramatically suppressed hepatic GNG at the level of key regulatory enzymes such as pyruvate carboxylase, phosphoenolpyruvate carboxykinase and glucose-6-phosphatase [6]. This downregulation of GNG was additionally confirmed by marked hypoglycemia. As a result, a progressive decline in the glucose level occurred initially in the liver, then in the blood and ultimately in the brain. However, only a twofold drop in the brain glucose level promoted a pathological cascade mediated by hyperactivation of NMDAR [51]. Under these conditions, an energy crisis developed in the brain preceding the appearance of convulsion, irreversible coma and death of the animal (Figure 1) [6].

**Figure 2:**
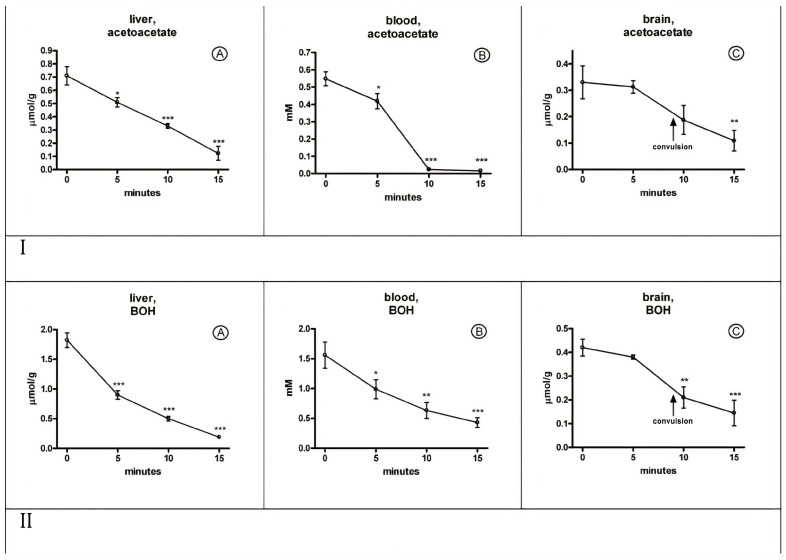
Time course of changes in acetoacetate (**I**), 3-hydroxybutyrate (**II**) in the liver (**A**), blood (**B**), brain (**C**) in acute ammonia intoxication. Values are the means ± SEM (*n* = 8). Significant differences between the values with respect to the time point of 0 min were estimated by ANOVA analysis and corrected with Bonferroni: * *p* < 0.05, ** *p* < 0.01, *** *p* < 0.001. This figure is taken from an original article “A Look into Liver Mitochondrial Dysfunction as a Hallmark in Progression of Brain Energy Crisis and Development of Neurologic Symptoms in Hepatic Encephalopathy” by Kosenko E. et al., published in J. Clin. Med. 2020, 9, 2259; doi:10.3390/jcm9072259 [6].

Furthermore, it was also revealed that a hepatotoxic effect of ammonia was associated with a sharp suppression of ketogenesis in the liver of animals [118]. As a consequence, ketone bodies disappeared from the blood and, like glucose, became inaccessible to the brain [6], thereby rendering neurons more vulnerable to ammonia-induced hypoglycemic death (Figure 2).

However, despite these findings, it remains unclear whether disease progression and increased patient mortality are related to these hepatotoxic effects of ammonia, which cause irreversible hypoglycemia-induced disturbances in oxidative metabolism in the human brain [119]. Nevertheless, the data from animal models indicate that it is the acute depletion of liver functional reserve caused by ammonia that leads to the immediate shutdown of essential glucose production and detoxification pathways; these results shed light on the mechanisms underlying chronic liver disease. Liver functional reserve is partially preserved in patients with chronic hepatitis (Child–Pugh score), but rapidly declines after the development of cirrhosis [1,120]. While the Child–Pugh clinical classification cannot be directly applied to animal studies with acute ammonia intoxication, the presented data suggest the possibility that the transition from stable adaptation to metabolic failure that occurs during the development of chronic hepatic encephalopathy, which completely depletes the functional reserve of the liver of patients and leads to the development of acute hypoglycemia, may be associated with a complete suppression of glycogenolysis and GNG.

Furthermore, studies on animal models have clearly shown that cerebral symptoms, seizures, precoma, then coma and death as the final event in ammonia poisoning have a common cause of hepatic origin. The role of ammonia as a hepatotoxin in the development of encephalopathy, whether initially associated with liver diseases or not [121], currently remains disputable [11]. Nonetheless, we anticipate that discussions of this topic will deepen the understanding of the underestimated hepatotoxic role of ammonia in studying the mechanisms of hepatic encephalopathy, which remains one of the longest-standing unsolved biomedical problems [122].

## 4. Erythrocytes as the Essential Factor in the Pathogenesis of Encephalopathy: The Role of Ammonia and NMDA Receptors

Other critical factors that cause or aggravate disturbances in brain energy metabolism and precede encephalopathy are limited transport and delivery of oxygen to the brain [123].

Indeed, both CBF and CMRO_2_ [17] are profoundly depressed in patients with liver cirrhosis across all stages from minimal HE [124] to acute HE episodes [125] and chronic deep coma [126]. This consistent reduction indicates that overall cerebral oxidative metabolism may be reduced regardless of disease stage and severity [127]. However, the relationship between CMRO_2_, CBF, and changes in blood ammonia levels in patients with HE has not been extensively studied. In light of this, the mechanisms responsible for hypoperfusion and disturbances of brain oxidative metabolism remain poorly understood. As mentioned above, the erythrotoxic effect of ammonia was discovered over a century ago, but these findings are not taken into account in clinical settings [13].

Importantly, it has been shown that the concentration of ammonia in the erythrocytes of hyperammonemic animals was approximately one and a half times higher than that in plasma [5]. But coma in animals, which was preceded by hyperventilation and clonic seizures, developed only when the concentration of ammonia in the erythrocytes was ten times higher than in the control [43].

A dramatic increase in ammonia level in the erythrocytes led to the suppression of glycolysis. It was confirmed by a significant decrease in the activity of key glycolytic enzymes such as hexokinase, phosphofructokinase, pyruvate kinase, as well as glyceraldehyde-3-phosphate dehydrogenase and lactate dehydrogenase and a sharp fall in the concentration of ATP in the erythrocytes [44]. This disturbance of the primary energy pathway could have been caused by a shift in the acid–base balance due to increased transport of two forms of ammonia (NH_3_ and NH_4_^+^) into erythrocytes, stemming from NH_4_^+^-dependent acidification that replaced the alkalization triggered by the primary transport of NH_3_ into cells [128]. Suppressed glycolysis and a subsequent decrease in ATP content reduced the activity of Na^+^, K^+^-ATPase leading to inability of this enzyme to restore the ion gradient disrupted by the transport of both forms of ammonia into erythrocytes [129,130]. This, in turn, led to disruption of the regulation of cell volume and osmotic resistance, formation of stomatocytes which contributed to intravascular hemolysis and hemoglobinemia [44] and the loss of numerous other functional properties of erythrocytes.

The accumulation of ammonia in erythrocytes also inhibited the pentose phosphate pathway, which was one of the causes of oxidative stress, manifested by accumulation of hydrogen peroxide and a decrease in the activity of antioxidant enzymes [43].

Ammonia has also been shown to reduce the concentration of 2,3-diphosphoglycerate (2,3-DPG) [44], the primary effector of oxygen binding to Hb in erythrocytes. As a result, Hb becomes unable to release the required amount of oxygen to tissues even with normal arterial oxygen saturation and oxygen partial pressure [22]. The significant negative correlation (r = −0.854, *p* = 0.0016) between these parameters indicates that the more ammonia accumulates in erythrocytes, the higher the likelihood that Hb fails to release required oxygen to tissues despite normal arterial oxygen saturation and partial pressure [44]. Unfortunately, this evidence is still ignored in clinical practice, and 2,3-DPG remains the most underestimated blood parameter indicating low oxygen delivery to the cells [27].

Furthermore, NMDAR play a variable role, either blocking, minimally attenuating, or having no effect on ammonia-induced changes in erythrocytes parameters. In particular, it was observed that MK-801 (a potent non-competitive NMDAR antagonist) given 30 min before ammonia injection did not prevent accumulation of ammonia in erythrocytes of hyperammonemic animals. These findings confirmed that transport and accumulation of both forms of ammonia depend on many factors [128] and are mediated by canonical, established membrane transporters rather than NMDAR-dependent pathways. Concurrently, ammonia-induced alterations in erythrocytes such as inhibition of Na^+^, K^+^-ATPase, decreased 2,3-DPG concentrations, and the conversion of normal erythrocytes to stomatocytes, also occurred independent of NMDAR activation [44].

Along with that, the revealed ammonia-related accumulation of hydrogen peroxide in the erythrocytes of animals as well as the disturbance of the redox balance and decreased activity of antioxidant enzymes, underlying the progression of oxidative stress, were partially prevented by the NMDAR blocker MK-801. Only glycolytic flux, inhibited by accumulated ammonia in erythrocytes, was completely restored in the case of pretreatment of rats with MK-801. Restoration of glycolysis in the presence of MK-801 was also confirmed by the values of lactate, pyruvate, ATP, ADP, AMP concentration, energy charge, the NAD^+^/NADH ratio as well as the activities of glycolytic regulatory enzymes that returned to control levels, thus suggesting that the ammonia-induced inhibition of glycolysis was mediated by activation of NMDAR [43,44]. The factors responsible for such selective involvement of NMDAR in ammonia-induced disruption of energy metabolism, antioxidant status and morpho-functional properties of erythrocytes triggering disturbances of their rheological functions and oxygen capacity are currently unknown, thereby necessitating deeper exploration.

In general, all the above indicate that ammonia is able to accumulate in significant quantities in erythrocytes. So, it is possible to make a conclusion that erythrocytes can serve as a temporary ammonia repository and its presence in the blood may have a number of pathological consequences both for erythrocytes themselves and for the entire body as a whole. This may occur because this ammonia storage is extremely unstable, since the accumulation of ammonia in erythrocytes and its release into systemic circulation depend on numerous endogenous factors [131,132] and are unpredictable. Unpredictability of these processes as well as the presence of preanalytical and analytical errors in the ammonia testing procedure [133] lead to an inaccurate assessment of its concentration in blood plasma and, accordingly, to ambiguous conclusions regarding the role of ammonia in HE of cirrhotic [134,135] and non-cirrhotic etiology [136].

More importantly, this approach overlooks the fact that mature erythrocytes in humans (as well as in rats and mice) do not contain either glutamine synthetase or glutamate dehydrogenase, which in other cells and tissues are the only enzymes that are directly involved in detoxification of ammonia. That is what makes erythrocytes extremely vulnerable (up to lysis) to ammonia, which can cause serious metabolic disorders, including those associated with damage to the oxygen-carrying capacity of erythrocytes, leading to cerebral hypoperfusion and irreversible neurological disorders [59].

Although tissue oxygenation is performed by the tightly coordinated oxygen transport system—the lungs, heart, and blood, the erythrocytes are key players and the sole blood cells that carry and directly deliver oxygen to tissues [137]. Undoubtedly, a thorough study of metabolic/energy pathways in erythrocytes that modulate oxygen transport capacity holds promise for identification of an additional risk factor for the development of cognitive impairment [138] associated with hypoperfusion and multiorgan hypoxia [139] in patients with HE accompanied by HA. This is especially important for older adults, whose erythrocytes are characterized by age-related impairments in many biochemical processes occurring in membranes and within these cells [61].

## 5. Conclusions

The relationship between liver damage and brain dysfunction (HE) has been known for over a century but the pathogenesis of this neuropathology has not yet been fully elucidated. Currently, brain injury is usually considered solely from the point of view of the neurotoxic effects of ammonia, causing an energy collapse of the brain, which underlies progressive neurodegeneration. Meanwhile, in recent years, there has been growing evidence that ammonia may have harmful effects on brain function in more ways than just directly.

**Figure 3:**
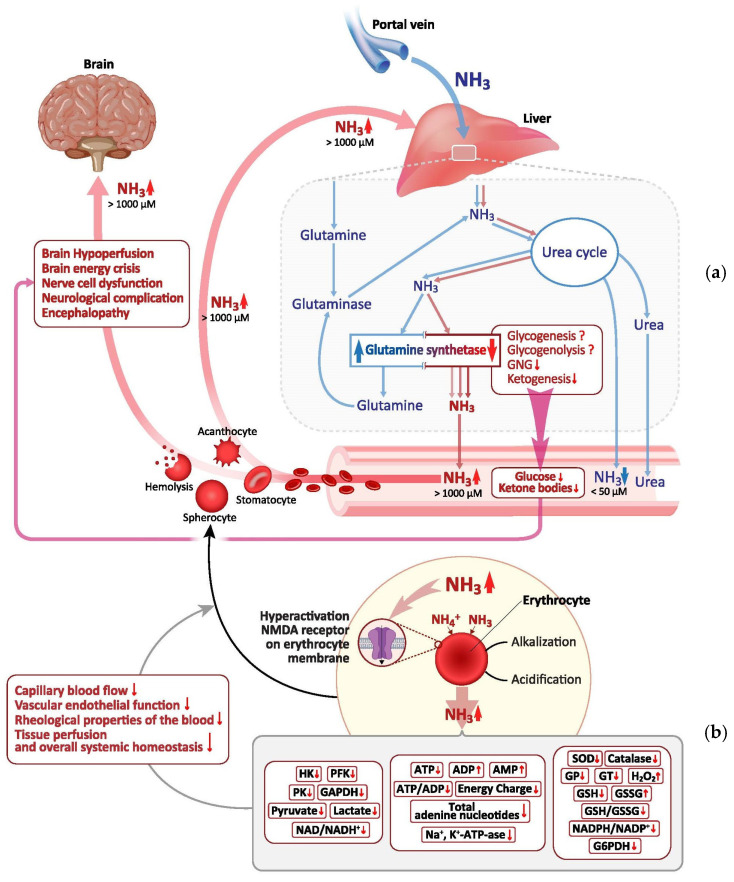
Ammonia-related disturbances of interorgan metabolic communication leading to a restriction of vital energy substrates to the brain. (**a**)—Toxic effect of ammonia on the liver. Blue arrows indicate the detoxification of ammonia in the liver under normal conditions, red arrows indicate impaired liver detoxification function, accumulation of ammonia and its toxic effect on cells. (**b**)—Toxic effect of ammonia on the erythrocytes. Abbreviations: G6PDH—glucose-6-phosphate dehydrogenase, GAPDH—glyceraldehyde-3-phosphate dehydrogenase, GP—glutathione peroxidase, GSH—glutathione reduced, GSSG—glutathione oxidized, GT—glutathione transferase, HK—hexokinase, PFK—phosphofructokinase, PK—pyruvate kinase, SOD—superoxide dismutase.

As a general conclusion, the data presented in this review indicate that besides the direct effect of ammonia on the brain, its toxic effect also spreads to the liver cells (Figure 3a) and erythrocytes (Figure 3b). Ammonia-induced damage renders these peripheral cells unable to provide vital energy substrates for the brain, thereby disrupting energy homeostasis and contributing to cerebral dysfunction.

The established role of ammonia in the disturbances of metabolic pathways in the liver and erythrocytes supports the hypothesis that the pathogenesis of HE is multifactorial.

Moreover, the summarized results demonstrate that NMDAR serve as a critical target for toxic effects of ammonia on the cells.

However, it should be emphasized that the intracellular NMDAR signaling cascades, involved in ammonia-induced disturbances of metabolic pathways in the liver and erythrocytes, are not uniform. The precise factors responsible for such selective NMDAR involvement in the ammonia-related disruption of energy metabolism, antioxidant status and morpho-functional properties, leading to dysfunction of the studied cells, are currently unknown and require further scrutiny.

Given that most of the blood ammonia enters erythrocytes, the poor correlation frequently observed between blood ammonia levels and HE severity at any given moment may be attributed to the unpredictable release of ammonia from erythrocytes, the levels of which in these cells in patients with HA is always several times higher than in plasma. Therefore, to prevent artifactually high or low results, ammonia levels in patients should be clinically monitored simultaneously in both plasma and erythrocytes.

The question of which is primary: the direct or indirect toxic effects that ammonia exerts on the brain, mediated by pathological disruption of the highly integrated “liver-erythrocyte-brain” metabolic axis, lies beyond the scope of this review. It is clear that both mechanisms are inextricably linked. Many aspects of these mechanisms are still unclear and require further study. However, it is obvious that to achieve a complete understanding of the true causes of HE, ammonia-related changes in the brain should be considered together with profound effects of ammonia on both the liver, which produces vital metabolites for the brain, and the oxygen-carrying function of erythrocytes, the sole cells that directly deliver oxygen to the tissues.

This approach will not only identify a key risk factor predisposing to the development of encephalopathy, but will also provide a unique opportunity to develop innovative personalized therapeutic strategies that could help address ammonia-induced metabolic disturbances in erythrocytes associated with cerebral hypoperfusion. Such safe and effective methods for reducing ammonia levels, based on the encapsulation of ammonia-detoxifying enzymes within erythrocytes (“ammocytes”, or “mini-livers”), have already been successfully tested in animal models of hyperammonemia.

## Data Availability

No new data were created or analyzed in this study. Data sharing is not applicable to this article.

## Abbreviations

The following abbreviations are used in this manuscript:

2,3-DPG	2,3-Diphosphoglycerate
CBF	Cerebral blood flow
CMRO_2_	Cerebral metabolic rate of oxygen
G6PDH	Glucose-6-phosphate dehydrogenase
GAPDH	Glyceraldehyde-3-phosphate dehydrogenase
GNG	Gluconeogenesis
GP	Glutathione peroxidase
GS	Glutamine synthetase
GSH	Glutathione reduced
GSSG	Glutathione oxidized
GT	Glutathione transferase
Hb	Hemoglobin
Hb-O_2_ affinity	Hemoglobin-oxygen affinity
HE	Hepatic encephalopathy
HK	Hexokinase
NMDAR	NMDA receptors
ODC	Oxygen dissociation curve
PaO_2_	Partial pressure of oxygen
PFK	Phosphofructokinase
PK	Pyruvate kinase
RDW	Red blood cell distribution width
SaO_2_	Arterial blood oxygen saturation
SOD	Superoxide dismutase
